# The Utility of Gallbladder Absence on Ultrasound for Children With Biliary Atresia

**DOI:** 10.3389/fped.2021.685268

**Published:** 2021-06-29

**Authors:** Andrea Ho, Marla A. Sacks, Amita Sapra, Faraz A. Khan

**Affiliations:** ^1^Department of Pediatrics, Loma Linda University Children's Hospital, Loma Linda, CA, United States; ^2^Division of Pediatric Surgery, Department of Surgery, Loma Linda University, Loma Linda, CA, United States; ^3^Division of Pediatric Radiology, Department of Radiology, Loma Linda University, Loma Linda, CA, United States

**Keywords:** abdominal ultrasound, biliary atresia diagnosis, biliary atresia, absent gallbladder, neonatal ultrasound

## Abstract

**Background:** Biliary Atresia (BA) is congenital condition, where infant intra- and extrahepatic bile ducts become obliterated, leading to cholestasis, and cirrhosis if untreated. This study aims to assess the predictive measure of absent gallbladder on ultrasounds (US) performed in infants with cholestasis for diagnosing BA.

**Method:** After Institutional Review Board approval, retrospective chart reviews of 61 infants with cholestasis found 43 (70.5%) were diagnosed with BA. A pediatric radiologist provided interpretations of all ultrasounds in a blinded fashion. Statistical analysis was used to assess the utility of absence of gallbladder on US in predicting BA, confirmed intraoperatively.

**Results:** Absent gallbladder on US predicts absent gallbladder with 77% accuracy, 92% sensitivity, 73% specificity, PPV 43%, and NPV 97% (*P* < 0.001, Fisher exact test). To diagnose BA, absent gallbladder on US has 66% accuracy, 53% sensitivity, 94% specificity, 96% PPV, and 46% NPV (*P* < 0.001, Fisher exact test).

**Conclusion:** Sonographic gallbladder absence has high specificity and PPV, indicating utility for BA diagnosis; however, it is not useful for ruling out BA given its low sensitivity.

## Introduction

Biliary atresia (BA) is a congenital condition occurring in infants in which intrahepatic and extrahepatic bile ducts are obliterated, leading to cholestasis and eventual cirrhosis if untreated. Prior to the implementation of Kasai portoenterostomy and liver transplantation, BA was considered fatal with a 2-year mortality of 95% (1). A systematic review from Canada shows that after Kasai portoenterostomy, the 4-year survival rate of BA patients was 81%, with 60% requiring a liver transplant (2, 3). BA is relatively rare, with an overall incidence of 1 in 10,000–20,000 live births; however, it is the most common condition causing neonatal jaundice that requires surgery and the most common disease leading to pediatric liver transplantation (4–6).

Typically accepted diagnostic tools at our institution include imaging [abdominal ultrasound (US), Hepatobiliary IminoDiacetic Acid (HIDA) scan], immunochemistry (ALP, AST, ALT, GGT, MMP-7), and tissue sample (liver biopsy) (7). Patients with BA may demonstrate failure of contrast passage to the duodenum on HIDA scan, elevated MMP-7, elevated transaminases, and bile plugs in portal bile ducts on liver biopsy (8, 9). Some countries have employed nationwide screening with a stool color card (10, 11). This was implemented for the first time by Taiwan in 2004, later followed by Japan in 2012; the sensitivity for detecting BA *via* stool cards in Taiwan in 2005 was found to be 97.1% (12). Additionally, screening direct bilirubin of neonates shortly after birth has been shown to result in earlier detection and treatment (7). In infants with cholestasis who undergo abdominal US, absence of the gallbladder is predictive of BA, with a specificity of 99% (8). However, US findings are to some degree sonographer-operator dependent and radiologist-interpretation specific.

**Figure 1:**
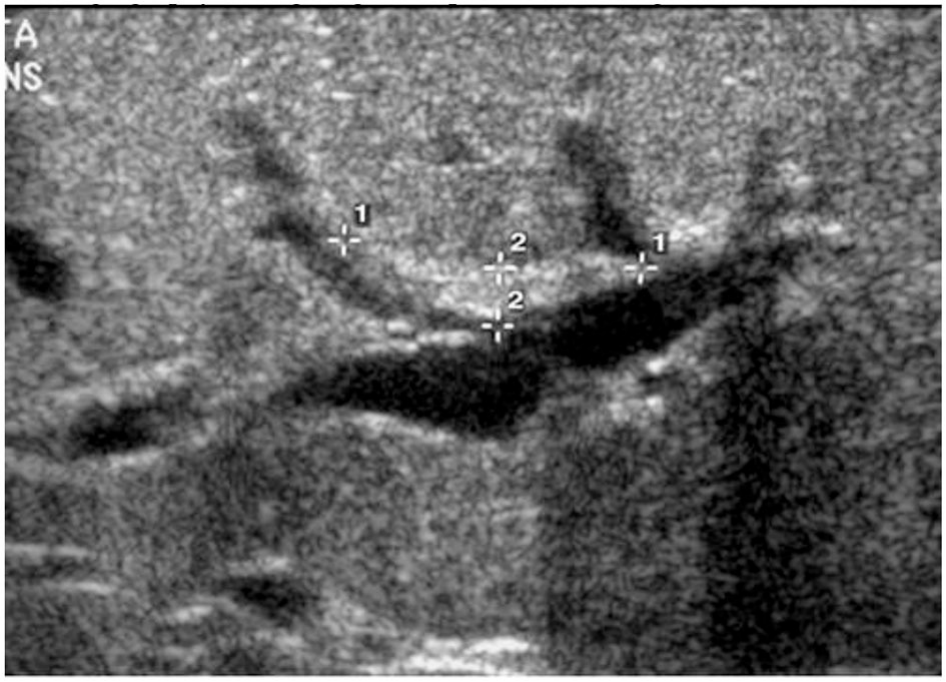
The triangular cord sign.

**Figure 2:**
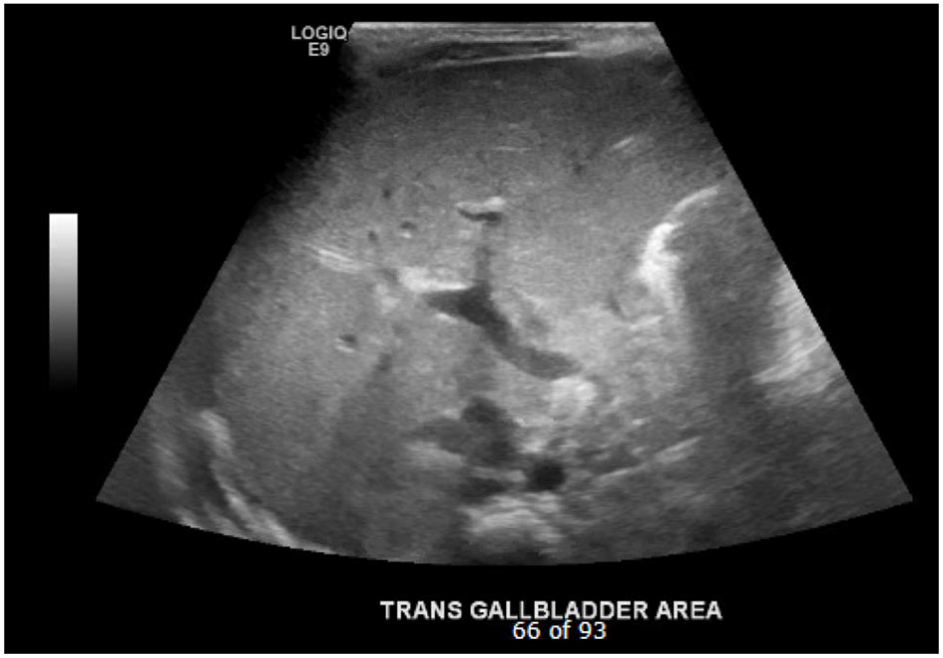
Absent gallbladder on ultrasound.

The triangular cord sign (Figure 1), which was first identified by Choi et al. in 1996 as representative of the fibrous cone at the porta hepatis, and gallbladder abnormalities (small gallbladder size with length <1.5 cm, abnormal shape and wall of gallbladder, and lack of gallbladder contraction after feeding) are two widely accepted US findings that suggest the diagnosis of BA in infants with cholestasis (4, 12–14). While several studies have analyzed and upheld the accuracy of a positive triangular cord sign in diagnosing BA, research has not similarly focused on the importance of gallbladder absence on US (Figure 2). The purpose of this study is to evaluate the utility of gallbladder absence seen on abdominal US in the diagnosis of BA, with absence of extrahepatic biliary tree on attempted cholangiography during surgical exploration as the gold standard.

## Materials and Methods

Approval was obtained from the Institutional Review Board (IRB #5200045, approval date February 4, 2020). A retrospective chart review was performed on 61 patients with elevated direct bilirubin levels who underwent abdominal US prior to abdominal exploration for suspicion of BA at a single institution. Fifty-four patients had abnormal HIDA scans indicative of BA; the other seven patients did not have records of HIDA scans. While the original diagnosis and decision for operation were made in an intradisciplinary fashion, all images were retrospectively reviewed by A.M., a pediatric radiologist, in a single-blinded process for the purposes of this study. Patients were excluded if variceal bleeding or hepatobiliary diseases other than BA were present. Abdominal USs and surgical procedures occurred between 2004 and 2019.

At our institution, infants, who are admitted for cholestasis, undergo abdominal USs and subsequent HIDA scans. Patients are not required to fast prior to abdominal US. If HIDA scan shows abnormal or no passage of the radiotracer from the gallbladder to the duodenum at 4 and 24 h, the test is considered to be indicative of BA. Consequently, patients undergo exploration for BA. Unless the biliary tree is absent, an intraoperative cholangiogram (IOC) is performed. Patients are diagnosed with BA, if the IOC shows no passage of contrast through the bile ducts or if the IOC was unable to be performed due to the degree of fibrosis present.

Statistical calculations were performed to evaluate the absence of a gallbladder on US as a predictive measure of discovering congenital gallbladder agenesis and BA confirmed during exploratory laparotomy. During surgery, gallbladders were considered absent if the surgical report indicated the following findings: completely absent gallbladder, fibrous remnant, obliterated, atretic, and completely fibrotic. Those that were normal in appearance, small, contracted, or only partially obliterated or fibrotic were considered present. Sensitivities, specificities, diagnostic accuracies, positive predictive values (PPV), and negative predictive values (NPV) were calculated, and 90% was the threshold used to determine significance.

## Results

Of the 61 patients, there were 32 females and 29 males. The patients' ages ranged from 1 day to 3 months at the time of abdominal US, with a median age of 8 weeks. At the time of exploratory laparotomy, ages ranged from 19 days to 6.5 months, with a median age of 8 weeks. In the case of the 1-day-old infant, an abdominal US was performed to evaluate for congenital anomalies in the context of congenital hydrocephalus, and the gallbladder was noted to be non-visualized. The infant later had worsening direct hyperbilirubinemia, and consequently concerns of BA were raised. Forty-three (70%) infants had BA confirmed by surgery, and 18 (30%) infants had BA ruled out by normal surgical and IOC findings, including normal appearance of gallbladder (Tables 1, 2).

**Table 1:** Sonographic comparison with intraoperative findings of gallbladder.

	**Gallbladder on laparotomy**	
**Gallbladder on ultrasound**	**Absent**	**Present**
Absent, *N* (%)	11 (18%)	13 (21%)
Present, *N* (%)	1 (2%)	36 (59%)

**Table 2:** Sonographic comparison with diagnosis of biliary atresia.

**Gallbladder on ultrasound**	**BA**	**No BA**
Absent, *N* (%)	23 (37%)	1 (2%)
Present, *N* (%)	20 (33%)	17 (28%)

Of the 12 infants with absent gallbladder during surgery, 11 (92%) had absent gallbladders on US, and one (8%) had visualized gallbladders on US. Of the 49 patients with gallbladders visualized during surgery, 13 (27%) had absent sonographic gallbladders and 36 (73%) had gallbladders visualized on US. The absence of gallbladder on US as an indicator of an absent gallbladder during surgery had a sensitivity of 92%, specificity of 73%, and diagnostic accuracy of 77% (Table 1). The PPV was 46%, and the NPV was 97%. With the Fisher exact test, the *p*-value was 0.0005, indicating that there is statistical significance (*P* < 0.01) between the outcomes of the group with absent gallbladder on US and the group with present gallbladder on US.

Of the 43 infants with confirmed BA, 23 (51%) had no gallbladder seen on US, and 20 (49%) had visible gallbladders on US (Table 2). Of the 18 infants who were not diagnosed with BA, one (6%) had no gallbladder on abdominal US, and 17 (94%) had visible gallbladders on US. Therefore, the sensitivity of absent sonographic gallbladder was 53%, the specificity was 94%, and diagnostic accuracy was 66% for diagnosing BA. The PPV was 96%, and the NPV was 46% (Table 2). The Fisher exact test resulted in a *p*-value of 0.001, which indicates that there is statistical significance (*P* < 0.01) between the outcomes of the group with absent gallbladder on US and the group with present gallbladder on US.

## Discussion

Non-visualization of the gallbladder during US has a sensitivity of 92% for ruling out gallbladder absence as determined by surgery; however, the poor sensitivity (53%) of the sonographic absence of a gallbladder for diagnosing BA indicates that it is a poor tool for ruling out BA. Therefore, even as non-visualization of the gallbladder in US may be somewhat useful for determining if the gallbladder is actually absent, the utility of this finding for ruling out BA is limited by the lack of clinical correlation with the diagnosis of BA. Of note, 13 of the 23 individuals with absent gallbladder on US had present gallbladders during surgery; this discrepancy is attributed to the majority of these 13 gallbladders being small, contracted, or intrahepatic during surgery. The difference between the sensitivities for the absence of gallbladder on surgery (92%) compared to the diagnosis of BA (53%) can be attributed to the number of patients who have gallbladders that are accurately detected on US yet nonetheless are ultimately diagnosed BA. Despite being a poor tool for ruling out BA, absent sonographic gallbladder has a high PPV (96%) and specificity (94%) for diagnosing BA. Therefore, if an infant has an absent gallbladder on US, there is a strong likelihood that the infant ultimately carries the diagnosis of BA.

The poor sensitivity of sonographic gallbladder absence for diagnosing BA in our study is consistent with the 28% sensitivity of gallbladder absence for BA diagnosis found by the meta-analysis by Zhou et al. (15). This is also in concordance with the finding that 20% of infants with BA may have normal gallbladders (16). The review by Zhou et al. included 23 studies published during 1998–2015 and identified the sensitivities, specificities, and diagnostic accuracies for BA diagnosis of various gallbladder abnormalities, the triangular cord sign, and hepatic artery enlargement. Gallbladder abnormalities included gallbladder absence, small gallbladder size with length <1.5 cm, abnormal shape and wall of gallbladder, and lack of gallbladder contraction after feeding (15). The US finding with the greatest sensitivity was a combination of triangular cord sign with gallbladder abnormalities (95% sensitivity), followed by the collective gallbladder abnormalities alone (85%), hepatic artery enlargement (79%), and triangular cord sign alone (74%). Amongst the gallbladder abnormalities, lack of contraction after feeding had the highest sensitivity (89%), followed by wall abnormality (83%), absence or length <1.5 cm (79%), and absence of gallbladder (28%). Interestingly, gallbladder absence on US had a specificity of 99% for diagnosing BA, which was higher than that of the triangular cord sign (97%), hepatic artery enlargement (75%), and the combination of triangular cord sign and gallbladder abnormalities (89%). Poor sensitivity, high specificity, and high PPV of non-visualized gallbladder on US for BA diagnosis is again demonstrated by Mittal et al., who reported that in infants under 90 days of age, absent gallbladder on US had a sensitivity, specificity, PPV, and NPV of 23, 100, 100, and 75%, respectively, for BA diagnosis (5, 17). Therefore, although non-visualization of the gallbladder on US is poor as an initial test for ruling out BA, it has consistently high specificity. This suggests that a clinician is likely to miss a diagnosis of BA if he or she is only relying on gallbladder absence on the US; however, in situations in which the gallbladder is absent on US, a BA diagnosis is highly probable.

The triangular cord sign is widely accepted as a highly reliable radiological finding in BA diagnosis. Takamizawa et al. proposes that when triangular cord sign is present, in conjunction with either a small gallbladder (length <1.5 cm or absent) or abnormal contractility of the gallbladder, ultrasonography alone can be used to diagnose BA (8). Thus, the triangular cord sign is useful as a standard of comparison for gallbladder absence. Gallbladder absence has similar specificity and PPV, but lower sensitivity and NPV, relative to the triangular cord sign. Whereas, the triangular cord sign has 74–84% sensitivity and 93% NPV, this study found that sonographic gallbladder absence has 53% sensitivity and 46% NPV (15, 18). However, the 98% specificity and 95% PPV of the triangular cord sign are similar to the 94% specificity and 96% PPV of sonographic gallbladder absence (15, 18). The poor sensitivity and NPV of gallbladder absence exclude it from being useful for BA, but if present, it is highly indictive of BA diagnosis and thus useful in the non-invasive diagnosis of BA.

Given its high specificity and PPV, non-visualization of gallbladder on US of an infant with cholestasis should incline a clinician toward the diagnosis of BA over other cholestatic conditions (19). In such infants, early exploration or at least close follow-up with serial imaging is strongly recommended. One important consideration is that gallbladder absence has in rare occasions been found in association with diseases other than BA, including duodenal atresia and choledocholithiasis (12, 20, 21). Thus false positive cases may occur, and clinical judgment should be used to determine if a concomitant condition is the more likely cause of non-visualized gallbladder.

One significant limitation of this study is the small sample size. Due to having a relatively low number of patients, the study may be limited by a lack of representative distribution of the population of individuals being evaluated for BA. Future research would benefit from including a larger number of patients. Another limitation of this study includes the usage of US as the “gold standard” in the field. Others have reported that there is variability of sonographers' skills (15), which can decrease the accuracy and, thus, the PPV. In addition, because infants are not required to fast prior to abdominal USs, it is possible that they had contracted gallbladders, which are smaller in comparison to non-contracted gallbladders and thus more difficult to visualize. Considering that every surgical procedure comes with risks, further research is needed to find non-invasive methods in BA diagnosis. Jancelewicz et al. (22) suggested in 2015 a diagnostic algorithm consisting of conjugated bilirubin and GGT levels, HIDA scan, abdominal US, and percutaneous cholangiogram for the diagnosis of BA, with negative laparotomy rate of 3–22%. If validated through prospective studies, this would be an efficient and relatively non-invasive means to diagnose BA. Future research in forming diagnostic algorithms would also benefit from combining the sonographic findings of absent gallbladder and triangular cord sign with other non-invasive diagnostic methods, such as MMP-7 levels. Given that there is considerable variation of utilizing liver biopsies amongst institutions, it would also be beneficial to study how these procedures in conjunction with absent gallbladder on abdominal sonography affect the accuracy of diagnosing BA.

## Conclusion

BA is a rare, once-fatal disease, characterized by obliteration of intra- and extrahepatic biliary ducts, that can lead to cholestasis and cirrhosis if untreated. It is thought that absence of a gallbladder on US is indicative of BA and thus could be useful as a diagnostic tool for ruling out BA. However, although an absent gallbladder on US has 92% sensitivity for predicting absent gallbladder, it has a poor sensitivity of 53% for predicting the diagnosis of BA. Thus, it is not a suitable tool for ruling out patients who may have BA. Despite its limited utility in ruling out BA, an absent gallbladder on US is highly predictive of BA as shown by a PPV of 96% and specificity of 94%. This is similar to PPV and specificity values of the triangular cord sign. In infants with cholestasis and non-visualized gallbladder on US, the diagnosis of BA along with surgical exploration should be strongly considered. In the future, research should be aimed at studying diagnostic algorithms and the utility of a combination of other investigations used for BA diagnosis, including liver biopsies and MMP-7 levels, in cases of non-visualized gallbladder on US. Additionally, further non-invasive modalities for diagnosing BA are needed.

## Data Availability Statement

The original contributions presented in the study are included in the article/Supplementary Material, further inquiries can be directed to the corresponding author/s.

## Ethics Statement

Written informed consent was obtained from the individual(s), and minor(s)' legal guardian/next of kin, for the publication of any potentially identifiable images or data included in this article.

## Author Contributions

AH participated in the study design, data entry, analyzed and interpreted the data, and wrote the initial draft of the manuscript. MS contributed to data analysis, interpretation of data, and assisted in drafting and editing the manuscript. AS reviewed all imaging and assisted in the radiological interpretation of our results. FK was involved study conception and design, analysis and interpretation of data arising, editing of the manuscript, and supervision of the project. All authors were involved in the critical review of the manuscript and all have approved and are accountable for the final version of the manuscript.

## Conflict of Interest

The authors declare that the research was conducted in the absence of any commercial or financial relationships that could be construed as a potential conflict of interest.
