# Supplementary material for: The Utility of Gallbladder Absence on Ultrasound for Children With Biliary Atresia
Source: Front Pediatr. 2021 Jun 29;9:685268. doi: 10.3389/fped.2021.685268 (PMC8275825; doi:10.3389/fped.2021.685268)
Supplement: Supplementary file 1 [file Table_1.DOCX]

**Appendix 1.** Patients with Biliary Atresia

| ID | Sex | Age at Ultrasound (Weeks) | Gallbladder Characteristics | Age at Surgery (Weeks) | Gallbladder Intraoperatively | Findings from Surgery |
| --- | --- | --- | --- | --- | --- | --- |
| P1 | F | 1 | Gallbladder present, minimally contracted | 2.5 | Present | Absent bile duct |
| P2 | M | 10 | Gallbladder small, contracted/septated/fibrotic | 11 | Present | Fibrotic/ absent bile ducts |
| P3 | M | 9 | No gallbladder | 10 | Absent | Unable to be performed due to no gallbladder lumen |
| P4 | F | 1 | Gallbladder present, contracted | 5 | Absent | Filling of biliary structure with no contrast extravasation |
| P5 | F | 5 | Gallbladder present, contracted | 6 | Present | No excretion through the bile ducts |
| P6 | M | 7 | No gallbladder | 8 | Present | Unable to be performed due to no lumen |
| P7 | F | 10 | Gallbladder present, contracted | 16 | Present | Contrast filled the residual lumen of the gallbladder but did not enter any ducts |
| P8 | M | 2 | No gallbladder | 7 | Present | No passage of contrast distally or proximally |
| P9 | F | 1.5 | No gallbladder | 2 | Present | No patent ducts, a small cystic structure filled with contrast |
| P10 | F | 10 | Gallbladder seen | 10 | Present | Choledochal cyst ended blindly in the porta hepatis |
| P11 | M | 12 | No gallbladder | 16 | Absent | Unable to perform |
| P12 | F | 12 | No gallbladder | 12 | Present | Not performed due to advance cirrhosis, ascites, and portal hypertension |
| P13 | M | 16 | Gallbladder present, contracted | 35 | Present | No filling of CBD seen |
| P14 | M | 9 | No gallbladder | 9 | Present | Unable to perform due to no lumen remaining |
| P15 | F | 11 | No gallbladder | 12 | Absent | No visible ducts on cholangiogram |
| P16 | F | 24 | Gallbladder small, contracted | 26 | Present | Not done due to cirrhotic changes and ascites |
| P17 | F | 8 | Gallbladder present, contracted | 9 | Present | Gallbladder lumen patent but cystic duct occluded |
| P18 | F | 2 | Gallbladder present | 4 | Present | Flow of contrast into the gallbladder but no evidence of cystic duct or biliary duct system |
| P19 | M | 10 | No gallbladder | 11 | Present | Not possible due to no gallbladder lumen |
| P20 | F | 10 | No gallbladder | 11 | Absent | No identifiable lumen for IOC |
| P21 | M | 11 | No gallbladder | 11 | Present | Patent gallbladder but no drainage into extrahepatic biliary system |
| P22 | F | 10 | No gallbladder | 12 | Absent | Not performed due to no gallbladder lumen |
| P23 | M | 8 | Gallbladder small, contracted | 9 | Present | There was filling of the distal common bile duct and filling of the duodenum.  However, further contrast injection did not demonstrate filling of the  superior hepatic ducts |
| P24 | M | 8 | No gallbladder | 8 | Present | Not performed as pathology analysis showed that the portal plate did not have any ducts |
| P25 | M | 1 | Gallbladder present, contracted | 4 | Present | Unable to clearly visualize intrahepatic bile ducts and CBD |
| P26 | F | 8 | No gallbladder | 8 | Present | Not possible due to no gallbladder lumen |
| P27 | M | 2 | Gallbladder present, contracted | 8 | Present | No evidence of patent ducts |
| P28 | M | 0.5 | Gallbladder small, contracted | 3 | Present | Attempted. However, gallbladder was totally obliterated |
| P29 | F | 7 | No gallbladder | 8 | Absent | Unable to be performed due to lack of gallbladder |
| P30 | F | 9 | Gallbladder present, contracted | 9 | Present | No evidence of ducts |
| P31 | F | 12 | No gallbladder | 12 | Absent | Not done due to presence of moderately cirrhotic liver |
| P32 | F | 6 | Gallbladder present, contracted | 8 | Absent | Distal bile duct was very thin, no filling of proximal duct |
| P33 | F | 8 | No gallbladder | 10 | Present | Failed due to no patent gallbladder lumen |
| P34 | F | 10 | No gallbladder | 10 | Present | No ductal filling |
| P35 | F | <1 | No gallbladder | 3 | Absent | Not done due to no gallbladder |
| P36 | F | 2 | No gallbladder | 6 | Present | No filling of any extrahepatic ducts |
| P37 | F | 8 | ? Gallbladder small, contracted | 8 | Present | No flow out of gallbladder, no evidence of biliary tree |
| P38 | M | 1 | No gallbladder | 20 | Absent | Not performed |
| P39 | M | 8 | No gallbladder | 8 | Present | Patent gallbladder with excellent filling to the distal bile ducts down into the duodenum, but no visualization of any proximal biliary tree |
| P40 | F | 7 | Gallbladder present, contracted | 7 | Present | No filling of ducts |
| P41 | M | 4 | No gallbladder | 7 | Absent | No ducts visualized with filling of contrast |
| P42 | F | 8 | Gallbladder small, contracted/fibrotic | 8 | Present | No filling of common/ proximal hepatic ducts, no emptying into CBD or duodenum |
| P43 | F | 4 | Gallbladder small, contracted | 8 | Absent | Filling of gallbladder but nothing distal to cystic duct |

**Appendix 2.** Patients without Biliary Atresia

| ID | Sex | Age at Ultrasound (Weeks) | Gallbladder Characteristics | Age at Surgery (Weeks) |
| --- | --- | --- | --- | --- |
| P44 | M | 8 | Gallbladder present | 9 |
| P45 | M | <1 | Gallbladder with sludge | 8 |
| P46 | M | 1 | Gallbladder small, contracted | 3 |
| P47 | M | 6 | Gallbladder small, contracted | 8 |
| P48 | F | 3 | Gallbladder small, contracted | 20 |
| P49 | F | 6 | Gallbladder small, contracted | 6 |
| P50 | M | 0.5 | Gallbladder small, contracted | 2 |
| P51 | M | 6 | Gallbladder small, contracted | 6 |
| P52 | F | 8 | Gallbladder contracted | 12 |
| P53 | M | 12 | Gallbladder contracted | 12 |
| P54 | M | 1.5 | Gallbladder small, contracted | 7 |
| P55 | M | 8 | Gallbladder small, contracted | 8 |
| P56 | M | 1.5 | No gallbladder | 4 |
| P57 | F | 10 | Gallbladder, contracted | 12 |
| P58 | F | 10 | Gallbladder, contracted | 13 |
| P59 | M | 16 | Gallbladder, contracted | 20 |
| P60 | F | 7 | Gallbladder, contracted | 10 |
| P61 | M | 10 | Gallbladder present | 10 |
